# Genomic data sharing in research across Europe: legal challenges and upcoming opportunities within the European Health Data Space

**DOI:** 10.1093/eurpub/ckaf070

**Published:** 2025-09-10

**Authors:** Romina Royo, Eva Garcia, Irene Schlünder, Christina Hilmarsen, Mette Kielsholm Thomsen, Emilie Cauët, Simon Kok Jensen, Simona Giardina, Aline Hebrant, Gordana Raicevic Toungouz, Marc Van Den Bulcke, Juan Arenas, Christian Fynbo Christiansen, Eivind Hovig, Petr Holub, Alfonso Valencia, Salvador Capella-Gutierrez

**Affiliations:** Life Sciences Department, Barcelona Supercomputing Center, Barcelona, Spain; BBMRI-ERIC, Graz, Austria; BBMRI-ERIC, Graz, Austria; TMF—Technologie- und Methodenplattform für die vernetzte medizinische Forschung e.V., Berlin, Germany; Norwegian Institute of Public Health, Health Data Service, Tynset, Norway; CONNECT—Center for Clinical and Genomic Data, Aarhus University Hospital, Aarhus N, Central Denmark Region, Denmark; Department of Clinical Epidemiology, Aarhus University Hospital and Aarhus University, Aarhus N, Denmark; Sciensano Cancer Centre, Brussels, Belgium; CONNECT—Center for Clinical and Genomic Data, Aarhus University Hospital, Aarhus N, Central Denmark Region, Denmark; Department of Clinical Epidemiology, Aarhus University Hospital and Aarhus University, Aarhus N, Denmark; Life Sciences Department, Barcelona Supercomputing Center, Barcelona, Spain; Sciensano Cancer Centre, Brussels, Belgium; Sciensano Cancer Centre, Brussels, Belgium; Sciensano Cancer Centre, Brussels, Belgium; ELIXIR Hub, Wellcome Genome Campus, Cambridge, United Kingdom; CONNECT—Center for Clinical and Genomic Data, Aarhus University Hospital, Aarhus N, Central Denmark Region, Denmark; Department of Clinical Epidemiology, Aarhus University Hospital and Aarhus University, Aarhus N, Denmark; Oslo University Hospital, Institute of Cancer Research, Oslo, Norway; BBMRI-ERIC, Graz, Austria; Institute of Computer Science, Masaryk University, Brno, Czechia; Life Sciences Department, Barcelona Supercomputing Center, Barcelona, Spain; Catalan Institution for Research and Advanced Studies (ICREA), Barcelona, Spain; Life Sciences Department, Barcelona Supercomputing Center, Barcelona, Spain

## Abstract

The European Health Data Space (EHDS) will help researchers use health data across EU Member States (MS). Currently, cross-border research faces heterogeneous data access processes. Using a real-world use case, this paper analyses challenges and opportunities brought by the upcoming implementation of the EHDS, assessing the situation before and after the regulation comes into force. The use case focused on metastatic colorectal cancer, analysing the relations between mutational signatures and clinical trajectories while addressing data access procedures across MS. The regulatory landscape and the challenges that need to be addressed for the EHDS to enable the secondary use of health data, particularly genomic data, are complex and heterogeneous across MS. We describe the pathway from data application to access to pseudonymized data in secure processing environments, emphasizing the legal requirements, including the role of ethics committees. Finally, we analyse the success factors for achieving access to the data and the reasons for access denial to support shaping the upcoming EHDS implementation. Several challenges remain unaddressed for cross-border data use, especially in the context of genomic data, where the complexity and heterogeneity of informed consent can impact or even impede data-sharing efforts. While EHDS can simplify processes across MS, it is crucial to ensure that additional safeguards do not negatively impact or block access to health data and that EHDS infrastructure is ready for effective and affordable processing of large volumes of genomic and other data.

## Introduction

The European Health Data Space (EHDS) regulation aims to implement a novel homogeneous mechanism for accessing and using health-related data across the EU and associated Member States (MS) for primary use, e.g. patient care, and for secondary use, e.g. research and policy-making purposes. Technological developments, including one or more digital platforms, are needed to achieve the ambition set in this regulation. In 2022, the European Commission launched the HealthData@EU Pilot project to better understand the possibilities and barriers of using health-related data for research, especially regarding the technological developments needed to respond to the upcoming regulation. This publication describes and discusses the challenges and learnings of accessing genomics data within the proposed EHDS [1], using insights from a near real-world research project: the ‘Cancer Genomics Use Case’ of the HealthData@EU Pilot project. This use case explored current challenges and obstacles while highlighting potential improvements in genomic data access and usage under the upcoming EHDS regulation. At the same time, the outcomes of this project will contribute insights into real-world challenges requiring attention in the future implementation of the EHDS, underscoring the project's importance in shaping the regulation's development.

In this use case, the secondary use of the data generated in clinical practice and the reuse of research data enable addressing questions of current medical importance, such as the relationship between medical trajectories and mutational signatures [2] which reflect the imprints of multiple mutational processes and provide insights into tumour aetiology. Efficient and secure integration of clinical and genomics data remains key to personalized medicine; expanding our understanding of disease mechanisms and enabling more precise diagnostics and therapeutic strategies that may ultimately improve patient outcomes [3–6].

The Cancer Genomics Use Case (Supplementary Fig. S1) focuses specifically on colorectal cancer (CRC), which has one of the highest cancer disease incidences across the European population [7]. The use case starts with the assembly of retrospective cohorts from different partner organizations that can enable the validation of known gene signatures and the discovery of new ones and their association with additional factors, such as tumour location or age. In the use case context, each participating organization, based in a different European country (Norway, Denmark, and Belgium), managed its own data application, reflecting the process researchers would typically follow. At the same time, these organizations played the role of health data access bodies (HDAB) in EHDS to better understand the implications of such an important actor and pilot the infrastructure. Similarly, BBMRI-ERIC served as a model of a European Research Infrastructure capable of both centralized and decentralized hosting and providing patient data, with its international role within the EHDS as an authorized participant, processing the access application and providing a Secure Processing Environment to process the data.

The Cancer Genomics Use Case was conceived to utilize whole-genome sequencing (WGS) or whole-exome sequencing (WES) data with its associated clinical data [8]. However, access to WGS/WES data was not homogeneous, with some use case participants unable to identify accessible WGS/WES datasets in their jurisdiction, as was originally planned, due to either the lack of the legal bases or the low availability of WGS/WES data compared to gene panels in clinical routine practices. This does not imply a lack of WGS/WES data in research, while the challenge lies in clinical data availability for secondary use within the EHDS framework. Throughout the development of the use case, strategies were adapted to accommodate the available data, e.g. gene panels, and the constraints of accessing them.

This work explores the implementation of a pilot use case within the framework of the EHDS. It was designed to incorporate genomic data, recognizing its potential impact on research, and to address the challenges of accessing data generated in health care for its use in research.

## Methods

WGS, WES, and gene panel data are generated in both routine clinical care and research projects to detect genetic variants for diagnosis, treatment, and scientific research [9]. While WGS and WES are extensively used in research, they are less common in clinical settings, where gene panels are more frequently applied. In Denmark, only a few genes are routinely examined for CRC diagnosis. WGS is performed only in patients with disseminated CRC who no longer respond to standard care and are potential candidates for targeted treatment. Similarly, in the BBMRI-ERIC CRC-Cohort, the consensus data model covers mutations in relevant genes from 25 institutions from 12 European countries, assuming that WGS/WES data can be generated from available linked biological material stored in biobanks.

Therefore, the scientific question was adapted to the project’s available data and time constraints. The original question was designed to address the relationships between mutational signatures and clinical characteristics, such as tumour localization or age of the patient. The revised hypothesis simplified this analysis due to the limited genome coverage in the available genomic data from most, which precluded the identification of mutational processes occurring outside of the targeted genes. This shift focused on annotating and prioritizing clinically relevant variants based on executing the Personal Cancer Genome Reporter (PCGR) [10]. This tool inputs Variant Call Format (VCF) files [11] and uses cancer-specific databases to provide insights into variants’ pathogenicity and clinical value.

After re-defining the research question, each use case participant started the process of applying for data access. Three institutions are based in different European countries (Denmark, Belgium, and Norway), while the fourth is a European Research Infrastructure (BBMRI-ERIC). The individual protocols are summarized below, with more details in the Supplementary Material.

### Belgium

#### Available data for request

Small (50 genes) to medium (150 genes) panels are covered by the national healthcare system, while Comprehensive Genomic Profiling (CGP) is not. The PRECISION initiative [Belgian Society of Medical Oncology (BSMO), 2018] provides molecularly guided treatments for metastatic solid tumours that are eligible for systematic therapy. It established a database collecting genomic and clinical information, including the Registry of the next-generation sequencing (NGS) tests used in clinical routine (NGS convention project). Two additional studies, the Belgian Approach for Local Laboratory Extensive Tumour Testing (BALLETT) and the Genetic, Neo (GeNeo) referring to ‘novel techniques’, have expanded patient access to CGP.

#### Data access protocols

Sciensano, the Scientific Institute of Public Health in Belgium, owns the NGS data, while the BSMO owns the BALLETT and GeNeo data. Since they were collected in clinical trials, access is limited to participating institutions, including Sciensano. Requests were made to access the VCF files of the NGS convention project and BALLETT, while only reported variants are available for GeNeo. Data is stored in Sciensano (Healthdata.be) with a data transfer agreement already in place. BSMO only required additional information regarding the use case.

### Denmark

#### Available data for request

Routine genetic analyses include a few relevant genes. For disseminated cancer, the National Genome Center may perform WGS for targeted treatments. Additionally, WGS, WES, or gene panels may be collected in research projects. Genomic data can be linked to regional and national clinical and administrative data using Denmark’s personal identification number [12].

#### Data access protocols

Regional ethics committees (REK) may approve the secondary use of non-extensive genomic analysis without consent, but the Danish National Committee on Health Research Ethics (NVK) [13] approval is required for WGS. Reuse of genomic data for research is considered a ‘health data science project’ and requires a specific hypothesis; exploratory studies need renewed consent. Thus, we requested NVK clarification on whether this use case qualifies as method development without ethical approval or whether it should be approved as a research project.

### Norway

#### Available data for request

Tumour genetic analyses may be performed in routine clinical care or in clinical studies. Results are only available in the patient’s electronic patient record or within clinical study databases, and their use is consent-based and limited to approved purposes. Two clinical trials (IMPRESS and COMET) were identified for this use case.

#### Data access protocols

REK approval under the Health Research Act was required. A protocol outlining the reuse of clinical trial data was submitted to the REK and approved. However, strict conditions were imposed, including the requirement for new informed consent from living patients and their biological relatives (for deceased patients) and an infrastructure to manage clinically important findings, such as re-identifying individuals.

Legal and ethical questions about these requirements were escalated to the joint National Ethics Committee (NEM). If fully approved, the analysis would require curated data from the Cancer Registry of Norway, and an application would need to be sent to the Health Data Services, which is managed by the Norwegian Institute of Public Health.

### BBMRI-ERIC

#### Available data for request

The BBMRI-ERIC CRC cohort (CRC-Cohort) contains clinical data from over 10 000 cases across Europe, covering key mutations, microsatellite instability, mismatch repair gene expression and Hereditary Nonpolyposis CRC assessment [14]. Some countries provide additional data, such as panel sequencing (e.g. TSO500), WGS/WES or whole slide images.

#### Data access protocols

Requests follow BBMRI-ERIC and CRC-Cohort Access Policies [15, 16]: (i) Registration: verification of requester identity and affiliation. (ii) Data request: submission via the BBMRI Negotiator platform [17] detailing the project, required data, and ethical approvals. (iii) Access control and data delivery: requesters must prove compliance with regulations and ethical standards, including data protection and consent. Approvals are evaluated by the Access Committee, involving all contributing biobanks. After approval, a Data Processing Agreement (DPA) is signed, and data are provided within a Secure Processing Environment. (iv) Return of results: requesters are encouraged to report outcomes and integrate derived data into the BBMRI-ERIC infrastructure for future accountability. BBMRI-ERIC-hosted data are processed for research under GDPR’s informed consent basis. When BBMRI-ERIC participates in EHDS as an authorized participant, it will continue using this basis since the legal obligation for data holders does not apply to authorized participants.

## Results

**Figure 1.**
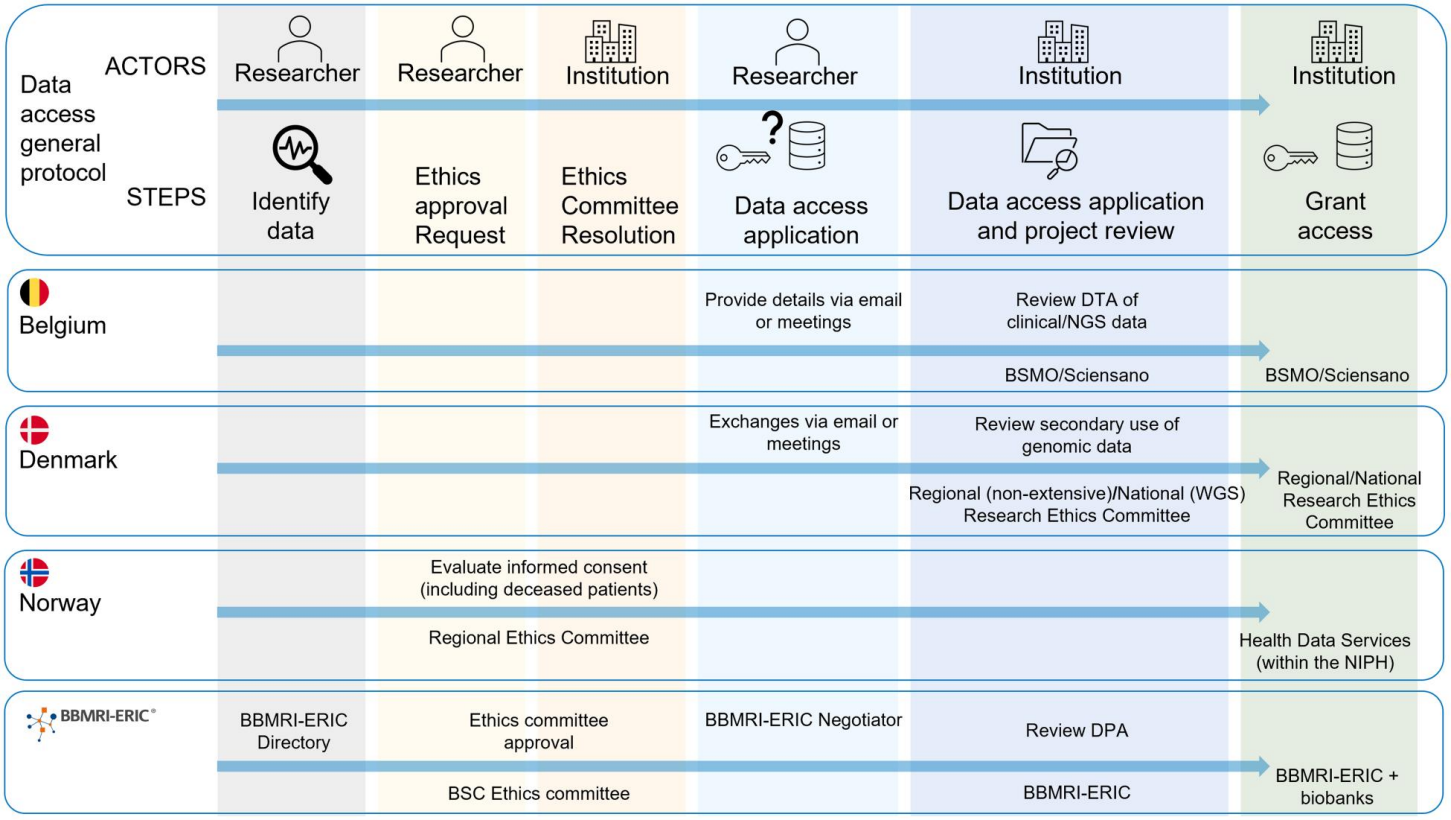
Implementation of the expected steps for data access protocols in each use case participant. Top, general overview (including those steps common for some partners) and (bottom) additional particularities of the steps and actors involved in the process for each partner. Of note, these data access protocols are usually followed by data processing (not shown). NIPH, Norwegian Institute of Public Health.

The Cancer Genomics Use Case was designed to use WGS/WES data as it provides extensive genome views [18, 19], which might contribute to confirming known signatures and potentially uncover new ones. However, we found limited WGS/WES data availability across use case participants, with minimal commonalities and different access protocols (Fig. 1). Some of them could access WGS/WES, while others only had gene panel sequencing data, ranging from comprehensive panels (300–500 genes) to very limited ones (<5 genes). Additionally, the Belgian partner could only provide tumour-only data, complicating the analysis due to the difficulty in distinguishing somatic mutations from artefacts and germline variants [20]. Based on this, we selected the most suitable datasets for the use case.

**Figure 2.**
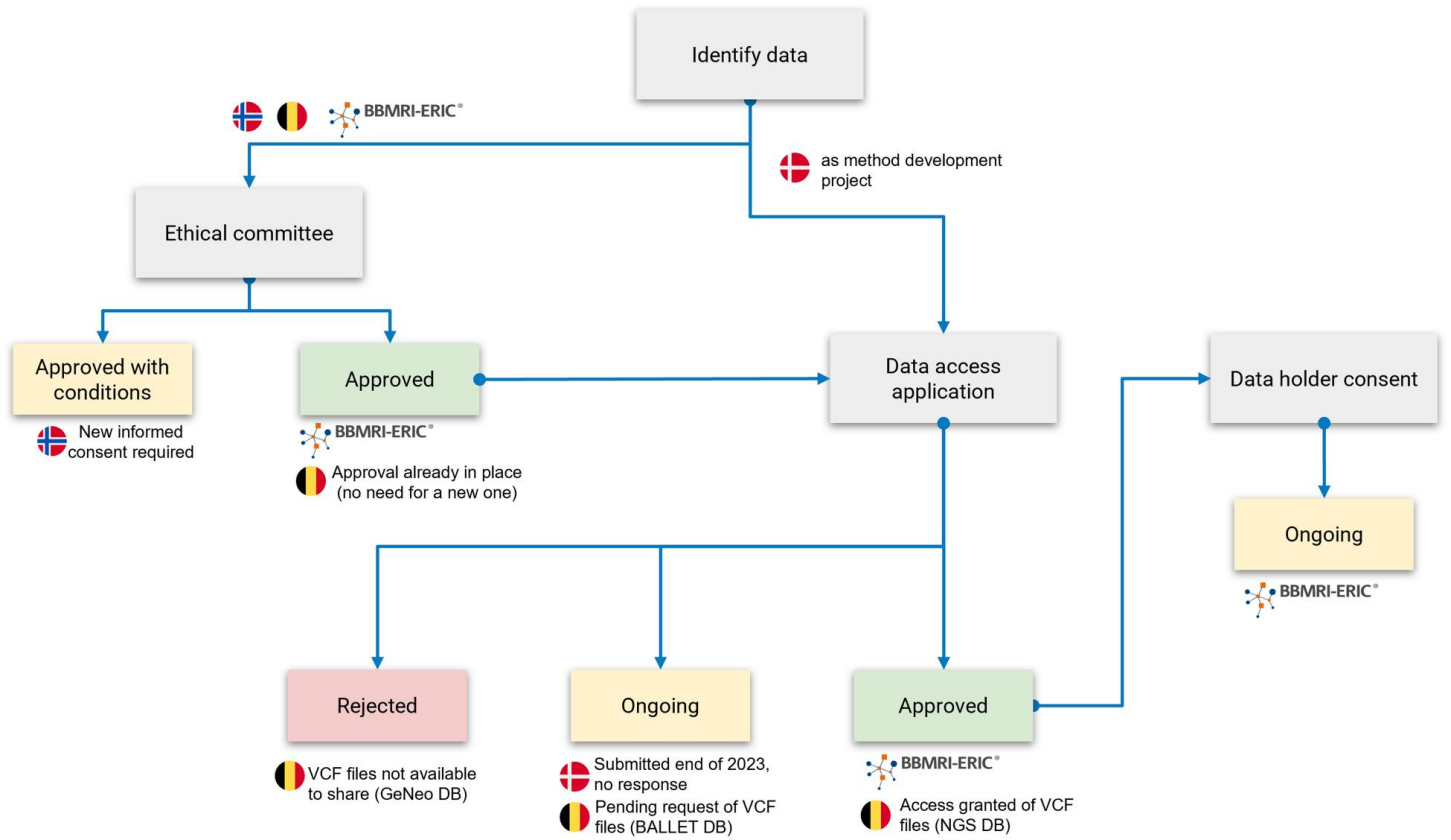
Comparison of data access steps and outcomes across use case participants. As of February 2025, the figure outlines the main steps for data access and their corresponding outcomes for each partner, represented by a country flag or organisation logo. In the case of the Danish organization, it was possible to select two different mechanisms to request access to the health data. The figure represents the path chosen as a method development project.

Beyond challenges in finding relevant data in the context of clinical data, the access processes across different EU countries, compared in Fig. 2, lacked clarity, requiring discussions with data owners, legal teams, and other authorities and resulting in imprecise and lengthy timelines for data access. The specific hurdles faced by each country highlight the variability in protocols across countries and research infrastructures, with different institutions enforcing differing levels of ethical approval. Two use case participants, BBMRI-ERIC and Norway, required ethical approvals but with different levels of stringency, while Denmark examined the option to apply for a method development project instead of a research project, focusing on the use case aim of assessing data access procedures across countries.

Just at the time of submitting this manuscript, the Belgium partner had managed to access the data, highlighting the challenges faced by all use case participants. The data access procedures were sometimes delayed due to lengthy discussions with regulatory bodies and ethics committees. In Denmark, nationwide genomic data has only become available for research in the National Genomic Database from June 2024, nearing the end of the use case period, and it was not possible to obtain all approvals and to get data access. A request was submitted to the National Research Ethics Committee at the end of 2023, proposing the project as a test of the EHDS setup, i.e. a method for European research projects. However, the committee did not respond in a timely manner, probably due to the request not fitting into the usual categories of applications. Similarly, in Norway, the REK required re-consenting, which was deemed both impractical and unethical, particularly for deceased patients where identifying next of kin was not feasible. It also required setting up a procedure for handling incidental findings, which was also deemed unethical, as contacting the closest relative (defined legally, not genetically) could cause several stressful reactions. This situation arose for the datasets that were selected based on having original consents for data sharing, thus implicating that in Norway, unless the consent is specific to the research question, it is not possible to have secondary use of clinical data with the current legislation.

Moreover, the complexity of obtaining data from multiple sources was exemplified in Belgium, where access was granted only to clinic-genomic data from the NGS study. Access to the VCF files for GeNeo was impossible due to restrictions imposed by Foundation Medicine (Roche FMI), the company conducting the testing, and data access for BALLETT remains pending.

Finally, the BBMRI-ERIC CRC-Cohort access procedure reflects the standard governance in European biomedical research, if not globally. A cornerstone in the access procedure is the submission of approval of the research protocol and the safeguards regarding the privacy of the participants by an independent Ethics Committee (EC) or Research Review Board by the applicant. Since there is no global standard for the required documents or the level of detail for research protocols, the approval procedures vary significantly, depending on the mechanisms in place at the institutions applying for data access, which can be lengthy, as in our case. After obtaining ethical approval, the Access Committee initiates the Access Procedure, which is approved or withheld by the data holders (contributors to the cohort) and can extend the data access timeline.

In summary, after >2 years, data were unavailable in most use case participants, as outlined in Table 1, which details the data requested at each of them and the current status. Our results illustrate the practical challenges and burdens currently faced by researchers when applying for access to health and genetic data. We observed that different data holders imposed varying requirements and protocols, which significantly impacted the efficiency of the data access process. Some data holders required additional documentation, while others had more stringent ethical approval processes that caused delays in access. All these insights will play a crucial role in shaping the upcoming implementation of EHDS. Indeed, it is expected that the implementation of the EHDS will contribute to alleviating the situation and providing consistency and clarity across the EU and associate MS.

**Table 1.** Table summarizing the datasets available for request at each use case participant

Participant	Dataset name	# Patients	Dataset data	Status
Belgium	NGS	∼3000	Small gene panel + clinical	Access granted to VCF files
Belgium	BALLET	59	Gene panel + clinical	Pending request for VCF files
Belgium	GeNeo	128	Gene panel + clinical	VCF files are not available to share
Denmark	Routine care WGS by the National Genome Center	∼5700 (only small subset CRC)	WGS + clinical	No response to the request as a Methods project
Norway	IMPRESS	18	Gene panel + WGS + clinical	REK required a new informed consent
Norway	COMET	70	Gene panel + clinical	REK required a new informed consent
BBMRI-ERIC	CRC-Cohort	∼400	WGS + clinical	Request approval from the data holder needed

## Discussion

This use case was set up to analyse current challenges in genomic data access, comparing them with the expected improvements after implementing the EHDS regulation. The practical implementation, based on the execution of the PCGR [10] to systematically analyse cancer genomes in the context of diagnostic, prognostic, and therapeutic biomarkers, showcased the challenges of accessing sensitive data, especially genomic data, and explored various possibilities across different settings, from national institutions to research infrastructures. The lessons learned from the entire process, in particular the ethical and legal aspects, should provide valuable input to projects that are supporting the development of the Implementation Acts by providing use cases and requirements that guide EHDS implementation, such as the Second Joint Action Towards the European Health Data Space (TEHDAS2, https://tehdas.eu/).

### Current situation of European cross-border access to genomic data

The first key challenge in the use case was the availability of WGS and WES data, particularly in health care, where such data are often available late in the course of the disease. Patients must survive long enough to benefit from WGS/WES-based diagnostics and have the potential benefit of such genomic profiling. This limitation led to a scarcity and heterogeneity of genomic data across countries. Taking into account the variety of genomics data available in each use case participant, ranging from very few genes to WGS, we opted for the most inclusive minimum common dataset possible, maximizing the participation of all involved partners in the use case and making every effort to preserve the fundamental aspects of the original protocols. We found that ethical permission for the secondary use of WGS/WES without new information is particularly difficult due to the increased risk of incidental findings of potential impact on the patients and their relatives [21], despite our focus on somatic variants. Removing germline (and rare) variants before making them available is an aspect to consider. However, this decision should be taken specifically for each study requesting access to WGS/WES data.

### How EHDS could change the landscape of data access across Europe

The EHDS will allow the use of data from hospitals, laboratories, and research institutions. This enormous amount of health-related data opens up many research opportunities, being complementary to those mechanisms that are already in place, such as informed consent or public interest as a legal basis, and data transfer agreements to transfer the data between involved parties Thus, the EHDS is an additional, not exclusive, legal basis for data access, and initiatives like GDI and EUCAIM support interoperability, ensuring multiple pathways for data reuse.

Thus, the EHDS will facilitate unified data discovery, as health data users must understand which data is available and where it is located. A European centralized metadata catalogue will be part of the HealthData@EU infrastructure, enhancing the findability of datasets and informing researchers about the quality and utility of the data through the label under development in the QUANTUM project (https://quantumproject.eu/). However, data might be extracted separately from various sources, integrated before becoming available (i.e. Belgium) and accessed in parallel across different countries. These operations are specific to each infrastructure and may incur costs and efforts, directly impacting the sustainability of data exploration and access models. Nonetheless, the EHDS partially covers costs through permit issuing and data provision fees. Without such a framework, ensuring long-term sustainability would be significantly more challenging. Regarding data access, it will be important to develop common protocols and evaluation criteria that are then applied by the different data access authorities. Even if those aspects are solved, problems of heterogeneity and syntactic and semantic interoperability will have to be addressed by using common metadata, data standards, ontologies and aligning terms so they share the same meaning.

Considering the massive investment needed to make legacy data fully interoperable without a clear use, a pragmatic solution could be to adopt interoperability mechanisms prospectively so that newly generated data would be interoperable across systems and countries by design. Retrospective data, which is very relevant for research, could be transformed—ideally at origin—when its secondary use makes it necessary.

Beyond having common data access protocols and clear interoperability mechanisms, it is crucial to consider multidimensional data linkage. Synchronization procedures between countries should enable that any health-related data point at the record level is assigned unequivocally to the same individual regardless of where the data were generated and/or captured. This represents a newer challenge that goes beyond secondary use and the organization of the national health systems, including public and private care [22].

Even though the access to health data for secondary use will be somehow harmonized, as shown in Table 2, the EHDS Regulation [23] does not address the main hurdle: the lack of streamlining of the formalities necessary in each country to qualify for a data access application. Furthermore, legal fragmentation can be more pronounced for genetic/genomic data due to additional safeguards that can be implemented in each country (Art 51 §4 of EHDS). While a general opt-out for citizens was agreed upon in the European Parliament at the last minute, the possibility was created for MS to provide special rules for these data, which in France, for example, consist of obtaining informed consent. Ultimately, this can lead to the same difficulties arising in the future that have burdened this use case: Does the existing consent cover the new use? How broad can consent be? Are there exceptions in national law? How do local ECs interpret existing consent and assess availability? Will they demand to go back to all research participants to obtain re-consent? What if patients have died in the meantime?

**Table 2:** . Comparison of relevant steps and actors for data access in the current project (pre-EHDS) and in the upcoming EHDS (post-EHDS)^a^

	Pre-EHDS		Post-EHDS	
	Actor	Action	Actor	Action
Identify data	U	Catalogue of each country or even institution	U	**Central catalogue**
Ethical approval request	U	Submit a project to EC	U	*Submit project to EC depending on factors, e.g. publishers requirements, funding bodies, etc.*
Ethical committee resolution	I	EC decision delivered	I	EC decision delivered
Data access application	U	Different request access protocols in each institution	U	**Unified request access through the central catalogue, that reaches the different HDABs**
Data access application and project review	I	The different institutions are responsible even in the same country	I	**The data access application will be managed by the HDABs**
Grant access	I	The different institutions are responsible	I	**HDABs are responsible**
Data linkage	I	The different institutions are responsible (depending on consent, etc.)	I	**HDABs are responsible**

^a^Two types of actors are reflected in the table: Data user (U) and Institution (I). Bold indicates improvements by EHDS and italics indicate processes to be further defined when implementing the regulation.

The Cancer Genomics Use Case described here made a full attempt and encountered difficulties that need resolution in access, homogenization, and, most importantly, a single, standardized legal/ethical pathway for Europe. Implementing the EHDS could solve these challenges, promising to facilitate equitable access to genomic data while safeguarding individual rights. Nonetheless, it is essential that safeguards for genomic data do not significantly hinder the objective of streamlining access to such data so that any constraints should not obstruct the mechanisms established by the EHDS to facilitate this process. By explicitly linking the challenges identified in our use case with the opportunities presented by the EHDS, we underscore the EHDS transformative potential to streamline genomic data-sharing processes, which will potentially have implications for human health in Europe.

## Supplementary Material

ckaf070_Supplementary_Data

## Disclaimer

Key points

- Harmonization of data access requests for genomics data should include regulating and standardizing informed consent processes to ensure that patient rights are protected and that data sharing is permitted and complies with ethical and legal requirements across different jurisdictions.
- Common protocols, accurate provenance descriptions, metadata standards, and synchronization procedures across countries are essential to ensure seamless cross-border data usage and address data heterogeneity and semantic interoperability issues.
- A key issue with the current EHDS Regulation text is that it does not cover procedures for Ethics Committees or fully address legal fragmentation around genetic/genomic data. Member States can impose specific rules, which can continue to raise data access challenges.

## References

[1] Hussein R , Balaur I, Burmann A et al Getting ready for the European Health Data Space (EHDS): IDERHA’s plan to align with the latest EHDS requirements for the secondary use of health data. Open Res Eur 2024;4:160. 39185338 10.12688/openreseurope.18179.1 PMC11342032

[2] Alexandrov LB , Kim J, Haradhvala NJ et al, PCAWG Consortium. The repertoire of mutational signatures in human cancer. Nature 2020;578:94–101. https://www.nature.com/articles/s41586-020-1943-3 32025018 10.1038/s41586-020-1943-3 PMC7054213

[3] Rulten SL , Grose RP, Gatz SA, Jones JL, Cameron AJM. The future of precision oncology. Int J Mol Sci 2023;24(16):12613. https://www.mdpi.com/1422-0067/24/16/12613/htm 37628794 10.3390/ijms241612613 PMC10454858

[4] Allam M , Cai S, Coskun AF. Multiplex bioimaging of single-cell spatial profiles for precision cancer diagnostics and therapeutics. NPJ Precis Oncol 2020;4:1–14. https://www.nature.com/articles/s41698-020-0114-1 32377572 10.1038/s41698-020-0114-1 PMC7195402

[5] Martyn M , Forbes E, Lee L et al Secondary use of genomic data: patients’ decisions at point of testing and perspectives to inform international data sharing. Eur J Hum Genet 2024;32:717–24. https://www.nature.com/articles/s41431-023-01531-5 38528053 10.1038/s41431-023-01531-5 PMC11153578

[6] Loane M , Loane M, Garne E et al Building public trust and confidence in secondary use of health data in Ireland. Eur J Public Health 2023;33. 10.1093/eurpub/ckad160.302

[7] Long D , Mao C, Zhang Z et al Long-term trends in the burden of colorectal cancer in Europe over three decades: a joinpoint regression and age-period-cohort analysis. Front Oncol 2023;13:1287653. 38115907 10.3389/fonc.2023.1287653 PMC10728819

[8] Tjota MY , Segal JP, Wang P. Clinical utility and benefits of comprehensive genomic profiling in cancer. J Appl Lab Med 2024;9:76–91. 10.1093/jalm/jfad091 38167763

[9] van Putten J. , Koster R., Snaebjornsson P., et al 1249P whole genome sequencing-based cancer diagnostics in routine clinical practice: an interim analysis of two years of real-world data. Ann Oncol. 2023;34:S727. http://www.annalsofoncology.org/article/S0923753423031757/fulltext

[10] Nakken S , Fournous G, Vodák D et al Personal cancer genome reporter: variant interpretation report for precision oncology. Bioinformatics 2018;34:1778–80. 10.1093/bioinformatics/btx817 29272339 PMC5946881

[11] VCFv, BCFv. *The Variant Call Format Specification*. 2024. https://github.com/samtools/hts-specs (23 October 2004, date last accessed).

[12] Laugesen K , Mengel-From J, Christensen K et al A review of major danish biobanks: advantages and possibilities of health research in Denmark. Clin Epidemiol 2023;15:213–39. 36852012 10.2147/CLEP.S392416 PMC9960719

[13] Home | Danish Research Ethics Committees. https://researchethics.dk/ (23 October 2004, date last accessed).

[14] Umar A , Boland CR, Terdiman JP et al Revised Bethesda Guidelines for hereditary nonpolyposis colorectal cancer (Lynch syndrome) and microsatellite instability. J Natl Cancer Inst 2004;96:261–8. 10.1093/jnci/djh034 14970275 PMC2933058

[15] BBMRI-ERIC. *BBMRI-ERIC Policy for Access to and Sharing of Biological Samples and Data*. https://zenodo.org/records/1241061 (23 October 2004, date last accessed).

[16] BBMRI-ERIC. *Access Policies—BBMRI-ERIC*. https://www.bbmri-eric.eu/services/access-policies/ (23 October 2004, date last accessed).

[17] Reihs R , Proynova R, Maqsood S et al BBMRI-ERIC negotiator: implementing efficient access to biobanks. Biopreserv Biobank 2021;19:414–21. https://pubmed.ncbi.nlm.nih.gov/34182766/34182766 10.1089/bio.2020.0144

[18] Brlek Petar , Bulić Luka, Bračić Matea, et al Implementing whole genome sequencing (WGS) in clinical practice: advantages, challenges, and future perspectives. Cells 2024;13(6):504. https://www.mdpi.com/2073-4409/13/6/504/htm 38534348 10.3390/cells13060504 PMC10969765

[19] Bagger FO , Borgwardt L, Jespersen AS et al Whole genome sequencing in clinical practice. BMC Med Genomics 2024;17:39–16. https://bmcmedgenomics.biomedcentral.com/articles/10.1186/s12920-024-01795-w 38287327 10.1186/s12920-024-01795-w PMC10823711

[20] Tumor-only sequencing has limitations. Cancer Discov. 2015;5:OF17. https://aacrjournals.org/cancerdiscovery/article/5/7/OF17/5149/Tumor-Only-Sequencing-Has-LimitationsTumor-Only 10.1158/2159-8290.CD-NB2015-064 25929850

[21] Green RC , Berg JS, Grody WW, et al, American College of Medical Genetics and Genomics. ACMG recommendations for reporting of incidental findings in clinical exome and genome sequencing. Genet Med 2013;15:565–74. https://pubmed.ncbi.nlm.nih.gov/23788249/23788249 10.1038/gim.2013.73 PMC3727274

[22] Raab R , Küderle A, Zakreuskaya A et al Federated electronic health records for the European Health Data Space. Lancet Digit Health 2023;5:e840–7–e847. 37741765 10.1016/S2589-7500(23)00156-5

[23] Regulation-EU-2025/327-EN-EUR-Lex. https://eur-lex.europa.eu/legal-content/EN/TXT/?uri=OJ:L_202500327

