# Supplementary material for: Genomic data sharing in research across Europe: legal challenges and upcoming opportunities within the European Health Data Space
Source: Eur J Public Health. 2025 Sep 10;35(Suppl 3):iii25–31. doi: 10.1093/eurpub/ckaf070 (PMC12420906; doi:10.1093/eurpub/ckaf070)
Supplement: ckaf070_Supplementary_Data [file ckaf070_supplementary_data.docx]

**Supplementary figures**


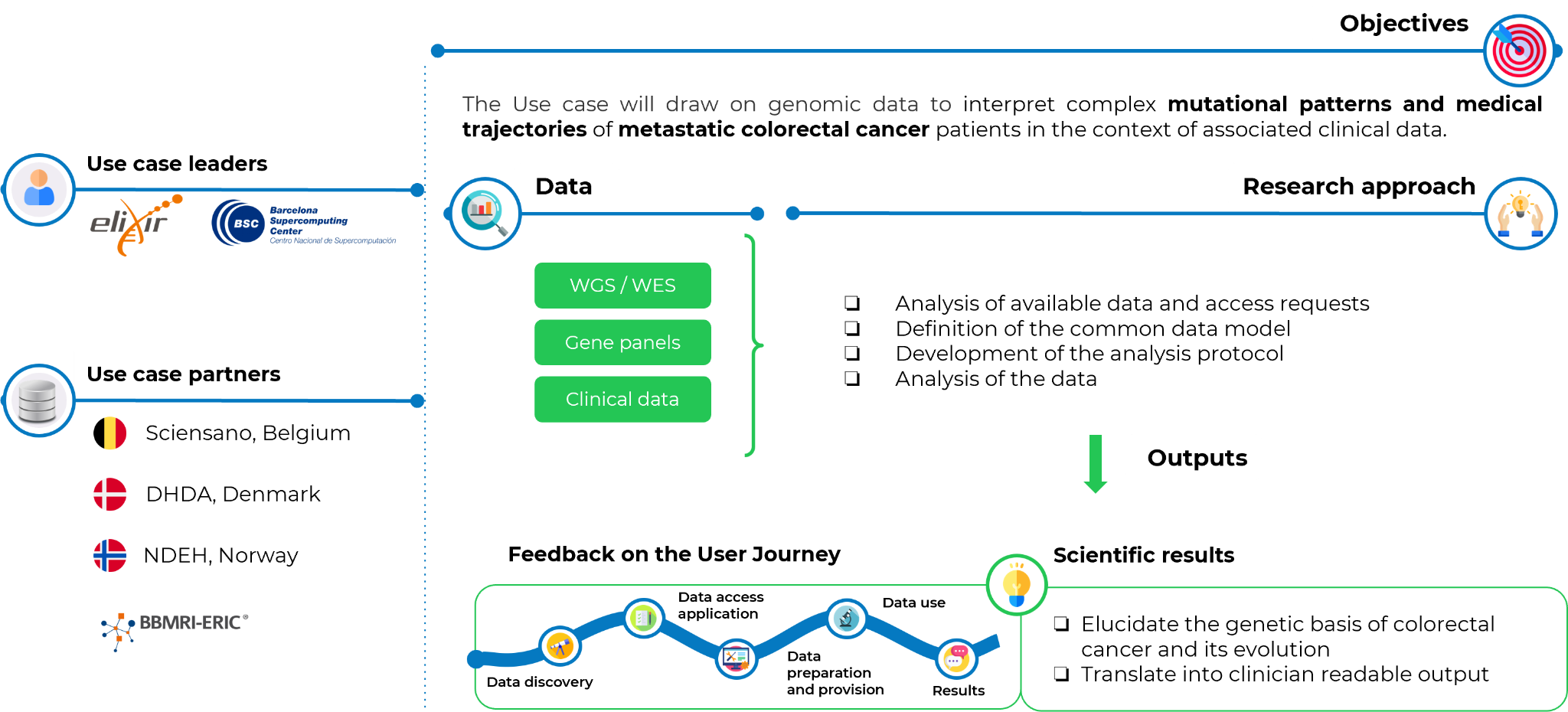


Fig. S1. **Original project plan of the Use Case**. Schema including the research team and use case participants (left), the objective and research plan, and the expected outputs at the scientific and operational levels, incorporating lessons learned from the process (right). WGS: whole genome sequencing; WES: whole exome sequencing; DHDA: Danish Health Data Authority; NDEH: Norwegian Directorate of eHealth.

**Supplementary methods**

The supplementary methods describe more in detail the protocols followed in each participating node.

**Belgium**

Next-Generation-Sequencing with small (50-gene) to medium (150-gene) panels is reimbursed only for specific indications covered by the national healthcare system. Comprehensive Genomic Profiling (CGP) is not currently reimbursed. However, cancer patients with metastatic locally advanced tumours can access CGP through clinical studies launched as part of the PRECISION initiative. The PRECISION initiative [1] was launched in 2018 by the Belgian Society of Medical Oncology (BSMO), in collaboration with various stakeholders. The aim was to correlate genomic and clinical data of patients with metastatic solid tumours that are eligible for systemic therapy across multiple Belgian oncology centres, with the ultimate goal to provide molecularly guided treatments for these patients. The PRECISION initiative has led to the establishment of a database, collecting both genomic and clinical information from patients. This includes the Registry Next-Generation-Sequencing tests for oncology (NGS). Additionally, two key studies, the Belgian Approach for Local Laboratory Extensive Tumour Testing (BALLETT) and the Genetic Neo, referring to ‘novel techniques’ (GeNeo), have increased the number of patients with advanced cancer that have comprehensive genotyping of their tumours. The database is stored in the Precision Belgium section of the Healthdata database, a national platform designed to collect and store the citizen’s health data in a secure and uniform manner.

Sciensano, the Scientific Institute of Public Health in Belgium, and a beneficiary partner in the EU project, holds ownership of the NGS data, while the BSMO owns the data from the BALLETT and GeNeo clinical studies. Since these data were collected within the context of clinical trials, access is currently limited to the participating institutions, including Sciensano. To contribute to the analysis phase of the current use case, the plan was to request access to the VCF (Variant Call Format) files of the three programs: NGS, BALLETT and GeNeo. Since the data are stored in the Healthdata platform (Sciensano) and that a Data Transfer Agreement (DTA) had already been signed between BSMO (the data owner) and Sciensano, BSMO only required additional information. These inquiries focussed mainly on specific aspects of the use case, such as data access and data security, and were provided through exchanges with BSMO representatives.

**Denmark**

Genetic analyses of tumours (typically a few relevant genes) are performed by pathology departments as part of routine clinical care. In patients with disseminated cancer, WGS of the tumour may be performed to identify potential genetic treatment targets. In this context, WGS is performed as a part of routine care by the National Genome Center in a project period and by the hospital owners (the Danish regions) afterwards. In addition, WGS, WES or gene panels may be collected as a part of research projects.

Regional research ethics committees may grant permission for secondary use of non-extensive genomic analysis with exemption from the consent requirement, but when whole-genome sequenced data are the subject of study, only the National Research Ethics Committee can grant permission for use of the data. Reuse of genomic data for research projects is considered a “health data science project” (distinct from a project requiring new genomic analysis, i.e. a “research project involving extensive mapping of an individual's genome, seeking exemption from the consent requirement”). Extensive guidelines are available for how to compile the application protocol. A central requirement is a very specific study question and hypothesis, and therefore explorative studies using genomic data do not fulfil the guidelines for being granted permission to reuse genomic data without renewed consent. The protocol of this use case was explorative in its nature, and did not provide sufficiently specific research questions to warrant a request to the National Research Ethics Committee. In the hope to gain access to data and contribute to the analysis phase of the use case, we posed a request to the National Research Ethics Committee, to define this use case as a test of the EHDS-setup, as a method for European research projects. Such a method test may not need an ethics committee approval, as it would not be a research project.

Genomic data can be linked to a number of regional and nationwide population-based clinical and administrative data using the unique personal identification number assigned to Danish citizens at birth or immigration [2].

**Norway**

Genetic analysis of tumours may be performed either as a part of routine clinical care, or as part of participating in an ongoing clinical study. As there is no central registry of genomic data, results are limited to the patient’s electronic patient record (EPR), and/or the clinical study databases with limited purposes and storage.

The results of genomic tests in clinical care are by regulation limited to use which may benefit the patient. Results from such tests in clinical trials are based on informed consent and limited to the purposes to which the patient has consented. In both categories, any use for other purposes - secondary use - is strictly limited. To participate in the use case, two eligible clinical trials (IMPRESS [3–5] and COMET [6]) were identified. These studies both included WGS data from CRC, as well as large gene panels (IMPRESS: TSO500, and COMET: Ion Torrent PGM with Ion AmpliSeq Cancer Hotspot Panel v2). In both studies, the informed consent was deemed open for reuse for a purpose such as in this use case. As the use case was deemed under the health research act, the project needed approval from a regional ethics committee (REK). An elaborate protocol was written to explain the Norwegian part of the use case, and the proposed re-use of clinical trial data.

The REK eventually approved [7] the project, but with strict conditions, effectively making the effort unattainable. They did not agree that given consents covered the suggested use in the use case, although both trials’ consents specifically mentioned better methods for diagnostics and treatment. Thus, planned re-use of the genetic data would require new informed consent from patients still alive, and informed consent from biological relatives of deceased patients. Also, an approval would require infrastructure designated to handle findings that might be of clinical importance to the individual, i.e., re-identify the individual, offering genetic guidance, treatment plans, etc. As the use case has an exploratory purpose within a limited cohort, the Norwegian team argued that any findings - positive as well as negative - would be highly unlikely. We argued that *biological* relatives differ from next of kin who may consent on behalf of the patient, following the Patient Rights Act. We also argued that retrieval of contact to biological relatives of those deceased would need to wash the population lists from the eligible studies towards the Population Registry, then identify biological relatives of the deceased in the Medical Birth Registry as biological children, siblings, parents etc., and further look up the biological relative(s) in the Population Registry for their contact information. Also, we pointed out the ethical considerations concerning the potential grievance of being contacted on behalf of a biological relative, perhaps also unintentionally revealing non-biological siblings in the process. The decision from REK was that these were all matters for the use case, and not an issue for REK.

As the Norwegian team questions the legal basis of requiring consent from *biological* relatives versus next of kin, and the requirement of follow up in case of findings, these issues have been raised before the joint national ethics committee (NEM). The experiences are also fed to an ongoing investigation on whether and how to establish a national genetic database.

Further, should a REK approval have been granted, the use case would need data curated by the Cancer Registry of Norway. Such an application, with the REK approval, would be sent to the Norwegian authority on granting access to such data, the Health Data Services, organised within the Norwegian Institute of Public Health.

**BBMRI-ERIC**

BBMRI-ERIC is the European research infrastructure for biobanking bringing together all the main players from the biobanking field – researchers, biobankers, industry, and patients – to boost biomedical research. The colorectal cancer cohort (CRC-Cohort) from BBMRI-ERIC contains clinical data from over 10,000 cases from across Europe. In addition, for some cases it also includes whole slide images and genomics data. The latter is the data type relevant for this Use case, together with the clinical data. In particular, WGS data are available for about 400 samples in the dataset. As mentioned in the introduction, the centralised dataset contains information on basic most commonly tested mutations (KRAS/NRAS mutations on exons 2/3/4, presence of BRAF/PIC3A/HER mutations, microsatellite instability, and mismatch gene expression, complemented with an Amsterdam criteria assessment for HNPCC [8]. In some countries, additional data is available, either panel sequencing (e.g., TSO500) or WGS/WES. Due to availability of biological samples, genotyping or sequencing can be further done, provided that there is funding available (which was not the case of EHDS2Pilot project unfortunately, hence we could rely only on the pre-existing data in this use case).

The request procedure for accessing samples/data comprises the following steps, as described in the BBMRI-ERIC Access Policy [9] and in the Access Policy for the CRC-Cohort [10]:

Step-1 **Registration of requester**: BBMRI-ERIC verifies the identity of each requester and his/her institutional affiliation (employee status).

Step-2 **Request of samples/data**: A requester files a request for access to samples/data via the BBMRI-ERIC IT services. For this use case, the BBMRI Negotiator [11] was the platform used for filling the request. Each request must provide information about the approved/proposed research project including its expected properties and amount of samples/data and their anticipated use, as well as the destination of the samples (if different from the location of the requester), and its ethical approval status.

The ethical approval should be granted by an Institutional Review Board or equivalent bodies that provide an independent evaluation for ensuring ethical compliance, the safety of research subjects, and conformity with current laws and data protection regulations.

BBMRI-ERIC may either request refinement of the request or provide availability information to the requester. In compliance with the governing ethical principles, availability Information is treated as confidential by BBMRI-ERIC, i.e., it will not be disclosed to other providers. Requests are not used for any other purpose than assessing the availability of the requested samples/data and providing offers.

Step-3 **Access control & samples/data delivery**: After receiving adequate availability information, the requester follows up directly with BBMRI-ERIC in order to provide any additional information needed to assess whether access can be granted. As part of this process, BBMRI-ERIC must comply with the regulatory and ethical conditions (e.g., data protection regulations, assessment of compliance of the national legal basis which might be informed consent in most cases, the approved/proposed project, checking whether the amount of deployable/extraditable samples required is scientifically justified) and transfer liability to the requester by using Data Transfer Agreements (DTAs) as deemed appropriate. For controlling access to the dataset, BBMRI-ERIC has an Access Committee comprising all the contributing partner biobanks, in order to ensure the due data release approvals are in place, particularly when releasing pseudonymous (personal) data.

After receiving an access request, the Data Manager checks whether the access request conforms with the formal requirements: (a) the identity of the requester is known and their institutional affiliation is provided, (b) the request contains project description.

If the formal requirements are fulfilled, the medical expert assesses the relevance of the project to the scope of CRC-Cohort.

If the project is within the scope, the Access Committee performs an Ethics Check procedure (either Expedite or Full, depending on whether sufficient previous ethics vote has been provided).

If all the previous steps conclude successfully, the Access Committee asks all the contributing partner biobanks for veto of the release. If only a subset of the CRC-Cohort is requested, only those partner biobanks are contacted who actually contributed the data specifically subject to the release.

After the access is approved by the Access Committee established by BBMRI, BBMRI-ERIC signs a DTA with the requester for the use of the data for the particular project. The DTA also transfers liability on the requester to ensure due data security measures when processing the data. If needed, special provisions have to be defined and agreed on a case by case basis and made available to the requesters.

Step-4 **Return of results**: Reports on project outcomes need to be collected for accountability purposes regarding the utilisation of the BBMRI-ERIC infrastructure. Thus, it is encouraged to require the return of derived data from the requester and integrate this requirement into their biobank policy and the respective MTA/DTA.

**References**

1. Thouvenin J, Van Marcke C, Decoster L, Raicevic G, Punie K, Vandenbulcke M, et al. PRECISION: the Belgian molecular profiling program of metastatic cancer for clinical decision and treatment assignment. ESMO Open. 2022 Aug 1;7(4):100524. Available from: http://www.esmoopen.com/article/S2059702922001454/fulltext

2. Laugesen K, Mengel-From J, Christensen K, Olsen J, Hougaard DM, Boding L, et al. A Review of Major Danish Biobanks: Advantages and Possibilities of Health Research in Denmark. Clin Epidemiol. 2023;15:213. Available from: https://pmc.ncbi.nlm.nih.gov/articles/PMC9960719/

3. Taskén K, Russnes HEG, Aas E, Bjørge L, Blix ES, Ahlquist TC, et al. A national precision cancer medicine implementation initiative for Norway. Nat Med. 2022 May 1;28(5):885–7. Available from: https://pubmed.ncbi.nlm.nih.gov/35513529/

4. Helland Å, Russnes HG, Fagereng GL, Al-Shibli K, Andersson Y, Berg T, et al. Improving public cancer care by implementing precision medicine in Norway: IMPRESS-Norway. J Transl Med. 2022 Dec 1;20(1). Available from: https://pubmed.ncbi.nlm.nih.gov/35568909/

5. Puco K, Fagereng GL, Brabrand S, Niehusmann P, Blix ES, Steinskog ESS, et al. IMPRESS-Norway: improving public cancer care by implementing precision medicine in Norway; inclusion rates and preliminary results. Acta Oncol. 2024;63:379–84. Available from: https://pubmed.ncbi.nlm.nih.gov/38779911/

6. Fretland AA, Dagenborg VJ, Bjørnelv GMW, Kazaryan AM, Kristiansen R, Fagerland MW, et al. Laparoscopic Versus Open Resection for Colorectal Liver Metastases: The OSLO-COMET Randomized Controlled Trial. Ann Surg. 2018 Feb 1;267(2):199–207. Available from: https://pubmed.ncbi.nlm.nih.gov/28657937/

7. Project #2698255 - Europeisk helserom-pilot (EHDS) - Cristin. Available from: https://app.cristin.no/projects/show.jsf?id=2698255

8. Umar A, Boland CR, Terdiman JP, Syngal S, de la Chapelle A, Rüschoff J, et al. Revised Bethesda Guidelines for Hereditary Nonpolyposis Colorectal Cancer (Lynch Syndrome) and Microsatellite Instability. JNCI: Journal of the National Cancer Institute. 2004 Feb 18;96(4):261–8. Available from: https://dx.doi.org/10.1093/jnci/djh034

9. BBMRI-ERIC Policy for Access to and Sharing of Biological Samples and Data. Available from: https://zenodo.org/records/1241061

10. Access Policies - BBMRI-ERIC. Available from: https://www.bbmri-eric.eu/services/access-policies/

11. Reihs R, Proynova R, Maqsood S, Ataian M, Lablans M, Quinlan PR, et al. BBMRI-ERIC Negotiator: Implementing Efficient Access to Biobanks. Biopreserv Biobank. 2021 Oct 1;19(5):414–21. Available from: https://pubmed.ncbi.nlm.nih.gov/34182766/
